# A validation study of the Arabic version of the Warwick-Edinburgh Mental Well-being scale among undergraduate students

**DOI:** 10.1186/s40359-023-01443-5

**Published:** 2023-11-17

**Authors:** Kashef Zayed, Ehab Omara, Ali Al-Shamli, Nasser Al-Rawahi, Ahmed Al Haramlah, Asma A. Al-Attiyah, Badriya Al-Haddabi, Ali Al-Yarobi, Majid Al-Busafi, Khalifa Al-Jadidi

**Affiliations:** 1 Sultan Qaboos University, Muscat, Sultanate of Oman; 2 Suhar University, Suhar, Sultanate of Oman; 3 Qatar University, Doha, Qatar; 4 Hafr Albaten University, Hafr Albatin, Kingdom of Saudi Arabia

**Keywords:** Validation, Mental Well-being, WEMWBS, Depression, Need satisfaction, Need frustration

## Abstract

**Background:**

The main aim of this study was to assess the validity of the Warwick-Edinburgh Mental Well-being Scale (WEMWBS) and the short version of the Warwick-Edinburgh Mental Well-being Scale (SWEMWBS) and to evaluate the metric properties of both versions by using a sample of undergraduate students from three Gulf Cooperation Council (GCC) countries (Oman, Qatar, and Saudi Arabia).

**Methods:**

Six hundred ninety undergraduate students (340 M and 350 F; mean _age_ = 21.16 ± 2.44) from Oman, Qatar, and Saudi Arabia (*N*_*OM*_ = 238, *N*_*QA*_ = 215, *N*_*SA*_ = 237), voluntarily participated in this cross-section study. All of them responded to the WEMWBS, Basic Psychological Needs Satisfaction Frustration (BPNSFS), and Beck Depression Inventory (BDI-II). The methodology involved utilizing descriptive statistics to understand the data’s characteristics, internal consistency analysis for reliability, correlation analysis for convergent validity, confirmatory factor analysis to validate the scales, and measurement invariance testing to ensure cross-group comparability. Model fit indices were employed to gauge the goodness of fit.

**Results:**

The translated Arabic versions of the WEMWBS and SWEMWBS showed good reliability, with Cronbach’s alpha values of 0.867 and 0.772, respectively. The findings of confirmatory factor analysis asserted the one-factor solution to interpret the item variances of the 14-item WEMWBS and 7-item SWEMWBS. The WEMWBS and SWEMWBS also showed significant positive relationships with need satisfaction and negative relationship with need frustration, and depression. Moreover, the SWEMWBS showed partial scalar invariance across genders and countries, while the WEMWBS showed only partial metric invariance across the three countries and partial scalar invariance across genders.

**Conclusions:**

Our study highlights the appropriateness of both versions of the Warwick-Edinburgh Mental Well-being Scale (WEMWBS) in assessing the psychological well-being of Arab undergraduate students. The employment of these tools is strongly encouraged for the assessment of mental well-being within a comparable adult population.

## Background

Mental well-being is a broad concept indicating the state in which an individual feels comfortable, healthy, and happy during a period [1]. It’s a reflection of the state in which the individual realizes his or her abilities, copes effectively with life stressors, works productively, and contributes to providing services to his or her community; therefore, the well-being concept refers to the positive side of mental health, not just the absence of mental illness [2].

Positive mental health is recognized as a very important determinant of general health and coping effectiveness [3, 4]; thus, there is growing global demand for instruments for assessing mental well-being for evaluation research and clinical purposes and for all practitioners interested in measuring public mental health in different population samples.

Mental well-being describes an individual’s ability to utilize his or her potential capabilities in effectively adapting to his or her life conditions [5], while Ryan and Daci [6] suggested that mental well-being is the positive aspect of mental health that is associated with satisfying the individual’s three basic needs (autonomy, competence, and relatedness).

Many instruments worldwide have been developed to assess mental health based on different conceptualization backgrounds, among them Ryff’s Scale of Psychological Well-being (SPWB), which assesses psychological functioning [7]; the Positive and Negative Affect Scale (PANAS), which assesses subjective well-being [8]; the World Health Organization–Five index, which covers physical and psychological aspects of well-being [9]; and the Well-Being Questionnaire (WBQ), which mainly measures positive well-being, negative well-being, and energy of people with chronic illness [10].

Tennant et al. [11] developed an interesting measure that has drawn considerable attention worldwide and has become one of the favoured scales for assessing mental well-being in various populations. The term mental well-being is a complex structure that covers two perspectives; the first is the internal perspective, which includes self-happiness and life satisfaction, while the second is the external perspective, which includes psychological functioning [11].

The Warwick Edinburgh Mental Well-being Scale (WEMWBS) has two versions. The full version consists of 14 items focusing on the positive aspects of mental health. The shortened version of the scale consists of 7 items and relates more to psychological functioning rather than feeling; thus, it addresses mental well-being from a different perspective [12].

The two versions of the scale have been validated and used widely in different cultures all over the world and in various population groups, such as the general population [13], students [14], people with disabilities [15], and patients [16].

Recent research has explored the relationship between mental well-being, depression, need fulfillment, and need frustration, primarily through the lens of the theory of basic psychological needs (BPNT), which identifies autonomy, competence, and relatedness as fundamental psychological needs. When these needs are fulfilled, individuals tend to experience positive emotions and motivation [6].

Numerous studies have supported the link between the fulfillment of psychological needs and positive mental well-being (e.g. [17]), as well as the connection between thwarted needs and depressive symptoms (e.g. [18]). However, the existing literature offers conflicting information.

Several factors may explain these inconsistencies. Demographic differences, such as age, could impact the strength of the association between these variables, with stronger effects in younger individuals. Alternatively, individual differences in personality or coping strategies may influence the relationship (e.g. [19]).

Overall, the research on the connection between mental well-being, depression, need satisfaction and need frustration is still in its early stages. Nonetheless, it underscores the importance of these concepts in understanding human well-being. To generalize these findings, it is essential to conduct more research in diverse cultural contexts beyond Western societies.

Moreover, in the Arab context, there is a growing demand for using a validated Arabic version of the WEMWBS for adults that adheres to appropriate psychometric standards. This need led us to go through assessing the validity and reliability of the two Arabic versions of the WEMWBS in a sample consisting of undergraduate students from three Gulf Cooperation Council (GCC) countries, considering that this distinct demographic group facing unique challenges and stressors related to their academic pursuits. The results obtained from our research exhibit potential applicability and generalizability to other Arab societies, owing to the presence of shared cultural features prevalent across the Arab world. The presence of cultural commonalities, such as language, traditions, and values, establishes a basis that implies the possible applicability of our research findings outside the particular setting of the GCC.

We hypothesized that the two back-translated Arabic versions of the scale would fit a single-factor model. We also expected the WEMWBS to be positively correlated with satisfaction with basic needs and negatively correlated with dissatisfaction with basic needs and depressive symptoms. Finally, we expected that WEMWBS scores would distinguish between students according to the different countries they belong to; however, we did not anticipate gender disparities among these countries due to their shared cultural norms and values related to gender roles, mental health, and emotional expression. It is conceivable that these common cultural elements may mitigate any gender differences in mental well-being. Furthermore, considering the study’s focus on undergraduate students, their educational environments might exert a substantial influence, as universities in these countries equally endorse gender equality and offer mental health support.

### Objectives

This study seeks to achieve two objectives:

1. To assess the validity of the Arabic version of the Warwick-Edinburgh Mental Well-being Scale (WEMWBS) and the shortened version of the Warwick-Edinburgh Mental Well-being Scale (SWEMWBS).
2. To evaluate the metric properties of both versions by using a sample of undergraduate students from three GCC countries.

## Methods

### Participants

This cross-sectional research was conducted in March 2019 across three Gulf Cooperation Council (GCC) countries: Oman, Qatar, and Saudi Arabia. Our investigation focused on undergraduate students who were enrolled at four prominent universities—Sultan Qaboos University, Suhar University, Qatar University, and Hafr Albatin University. To ensure the participation of students, we implemented a comprehensive recruitment approach, encompassing email outreach, social media engagement, and direct contact within academic networks. We extended invitations to approximately 2,100 undergraduate students affiliated with these universities, inviting them to take part in our web-based survey.

Our dedicated efforts yielded a commendable response rate of 41%. Subsequently, we excluded incomplete surveys and all surveys answered by expatriate undergraduate students. This process resulted in a final sample size of 690 undergraduate participants, comprising 340 males and 350 females, with an average age of 21.16 ± 2.44 years. The distribution of this sample across the three countries was as follows: Oman (N = 238), Qatar (N = 215), and Saudi Arabia (N = 237).

In adherence to the highest standards of research ethics, the survey was meticulously designed to maintain anonymity, with no collection of personal data. Moreover, we secured informed consent from all participants, reaffirming our unwavering commitment to preserving the confidentiality of their data and utilizing it solely for research purposes.

### Materials

In addition to the demographic information, the study used four measures, all of which were self-evaluated:

#### Warwick-Edinburgh Mental Well-being scale (WEMWBS)

This tool was developed by a team of researchers from Scottish universities [11]. It consists of 14 positively phrased statements to assess mental well-being by using a 5-point response scale (*1 = none of the time to 5 = all of the time*). Therefore, the total score on the scale ranges from 14 to 70. Respondents must read each statement carefully and determine the extent to which it applies to them during the past two weeks. Thus, higher scale scores indicate higher levels of psychological well-being.

In 2012, the first author of this study translated the scale to Arabic after obtaining official approval from its authors. The internal consistency of the Arabic version of the scale reached 0.91 in the current study.

In 2009, Stewart-Brown et al. [12] developed a shortened version of the Warwick-Edinburgh Mental Well-being Scale *(SWEMWBS).* This version consists of seven items of the original WEMWBS’s 14 items, namely, items 1, 2, 3, 6, 7, 9, and 11. The total score of the SWEMWBS scale ranges from 7 to 35. In our current study, the internal consistency of the SWEMWBS was high (Cronbach’s α > 0.89).

#### Basic psychological need satisfaction–frustration scale (BPNSFS)

The validated Arabic version of the Basic Psychological Need Satisfaction-Frustration scale [20] was translated into Arabic by the guidance of the second edition of the International Test Commission for translating and adapting tests (following the guidance of the second edition of the International Test Commission guidance for translating and adapting tests [21]. The scale consists of two subscales: The first covers need satisfaction and consists of 12 items, while the second covers need frustration and consists of 12 items. The two subscales have three dimensions—autonomy, relatedness, and competence—derived from self-determination theory [6]. Official permission was obtained from the scale’s creators before translating it into Arabic. The internal consistency of the Arabic version of the BPNSFS varied from α > 0.81 [20] to α > 0.91 in our current study.

#### Beck Depression Inventory-II (BDI-II)

This scale is widely used to assess depressive symptoms. It consists of 21 groups of items representing all aspects of depressive symptoms. In each group, the respondent has to choose one of the four answers as follows: the first illustrates no depressive symptoms present, the second indicates that mild symptoms are present, the third indicates that medium symptoms are present, while the fourth answer illustrates that severe symptoms are present. The internal consistency of the Arabic version of BDI-II ranged from an average of α > 0.86 [22] to α > 0.85 in our current study.

### Procedures

Participants in the current study were recruited from universities in Oman, Qatar, and Saudi Arabia. countries. Paper questionnaires were distributed to the participants. The questionnaires included demographic questions and three instruments: the WEMWBS, BDI-II, and BPNSFS. The response period took approximately 20 min.

### Data analysis

Using SPSS 23, descriptive statistics, including means, standard deviations, skewness, and kurtosis, were calculated for the WEMWBS and SWEMWBS. We utilized skewness and kurtosis to determine whether the data were normal. When both findings fall within the range of + 2 to -2, normality is declared [23]. Cronbach’s alpha coefficients corrected item–total correlation, and the Spearman item correlation matrix was produced to evaluate the internal consistency of the complete and shortened versions of the scale. To evaluate how accurately the scale measures the outcome it was designed to measure, convergent validity was evaluated by investigating the correlation coefficients of the scores on the full and shortened scales with other related and well-established instruments. The need satisfaction, need frustration, and depression scales were all used to cross-check the original WEMWBS and SWEMWBS.

Furthermore, the constructor factorial validity of the WEMWBS and SWEMWBS was investigated using confirmatory factor analysis on the entire sample. Measurement invariance was carried out across countries and genders to ensure that the WEMWBS and SWEMWBS were comparable. To test measurement invariance and establish cross-group comparisons, a series of iterative processes were used. We looked at the construct’s stability across countries and genders through configurable invariance. Metric invariance was also used to assess the comparability of item factor loadings across countries and gender subgroups. Metric invariance compares the strength of the relationships between the WEMWBS and SWEMWBS items and their underlying components across groups. Scalar invariance was further examined by restricting item intercepts across country and gender subgroups to be equal. This level of scale measurement invariance is essential to compare the latent means among the intended study groups [24]. AMOS-20 was used for the confirmatory factor analyses. The model fit was investigated using the cut-off values for the following indices: Tucker‒Lewis index (TLI), comparative fit index (CFI; model fit is good when it exceeds 0.95 and acceptable when it exceeds 0.90) [25], and root mean square error of approximation (RMSEA; when values are less than 0.08, the fit is acceptable; when values are less than 0.05, the fit is good). RMSEA and CFI values less than or equal to 0.015 and 0.01, respectively, were employed as model comparison criteria [26].

## Results

### Descriptive statistics and internal consistency

The means, standard deviations, skewness, kurtosis, and corrected item–total correlations for each of the 14 WEMWBS items (N = 690) are displayed in Table 1. For the 14-item WEMWBS, the corrected item-to-total correlations varied from 0.438 to 0.594. It is worth noting that none of the items have severe skewness or kurtosis since all of the values are between 2 and − 2, indicating that all of the items’ responses are normal [23]. Furthermore, the Spearman correlation matrix table that shows the relationships between all 14 items is presented in Table 2. The majority of the correlation coefficients were greater than 0.300, which was suitable for factor analysis [27]. The WEMWBS and SWEMWBS Cronbach’s alphas were 0.867 and 0.772, respectively, which indicates good reliability. Additionally, the Cronbach alpha coefficients were comparable to the original Cronbach alpha value of the WEMWBS (0.890) and other relevant research [11, 28, 29]. The WEMWBS and SWEMWBS correlated with a Cronbach alpha value of 0.943 (*p* < 0.001), which was close to the 0.954 reported by the original developers [12].

**Table 1:** Descriptive statistics and reliability indices for the WEMWBS and SWEMWBS

Items	Mean	Std. Deviation	Skewness	Kurtosis	Corrected Item–Total Correlation	Cronbach’s Alpha if Item Deleted
MH1	3.90	1.158	-0.851	-0.134	0.522	0.859
MH2	3.96	1.095	-0.902	0.054	0.594	0.855
MH3	3.52	1.124	-0.395	-0.444	0.592	0.855
MH4	3.63	1.214	-0.509	-0.664	0.464	0.862
MH5	3.48	1.142	-0.406	-0.478	0.556	0.857
MH6	3.77	1.032	-0.600	-0.180	0.513	0.859
MH7	3.53	1.100	-0.389	-0.509	0.490	0.860
MH8	4.06	1.038	-1.009	0.498	0.584	0.856
MH9	3.72	1.112	-0.644	-0.184	0.499	0.860
MH10	4.09	1.025	-0.991	0.456	0.539	0.858
MH11	4.06	1.036	-1.055	0.642	0.512	0.859
MH12	3.89	0.986	-0.625	-0.106	0.495	0.860
MH13	4.12	1.014	-0.987	0.281	0.438	0.863
MH14	3.69	1.141	-0.656	-0.195	0.541	0.858

**Table 2:** Spearman correlation matrix for the WEMWBS items

Items	1	2	3	4	5	6	7	8	9	10	11	12	13	14
MH1	1.00													
MH2	0.47	1.00												
MH3	0.41	0.40	1.00											
MH4	0.27	0.32	0.29	1.00										
MH5	0.32	0.33	0.40	0.37	1.00									
MH6	0.21	0.30	0.32	0.24	0.45	1.00								
MH7	0.25	0.30	0.36	0.24	0.40	0.50	1.00							
MH8	0.36	0.45	0.46	0.24	0.28	0.40	0.34	1.00						
MH9	0.25	0.31	0.26	0.38	0.34	0.29	0.28	0.28	1.00					
MH10	0.37	0.40	0.36	0.27	0.24	0.30	0.26	0.45	0.29	1.00				
MH11	0.27	0.38	0.34	0.22	0.25	0.34	0.33	0.35	0.31	0.46	1.00			
MH12	0.31	0.34	0.30	0.36	0.24	0.23	0.29	0.32	0.37	0.34	0.34	1.00		
MH13	0.33	0.34	0.25	0.23	0.24	0.26	0.29	0.31	0.27	0.30	0.27	0.34	1.00	
MH14	0.33	0.34	0.48	0.27	0.48	0.33	0.27	0.38	0.35	0.24	0.24	0.26	0.30	1.00

### Convergent validity

The WEMWBS was found to have substantial moderate to high positive correlations with domains such as positive affect, life satisfaction, and overall health, as well as a significant negative correlation with symptoms of anxiety and depression in earlier investigations [11, 30–33]. This study reported the correlation between the overall WEMWBS and SWEMWBS, as well as the individual items and other construct-related measures (see Table 3). A significant positive correlation was observed with needs satisfaction (*R*_*WEMWBS*_ = 0.552, *R*_*SWEMWBS*_ = 0.544, *p* < 0.01) and a significant negative correlation with needs frustration (*R*_*WEMWBS*_ = -0.389, *R*_*SWEMWBS*_ = -0.359, *p* < 0.01) and depression (*R*_*WEMWBS*_ = -0.373, *R*_*SWEMWBS*_ = -0.337, *p* < 0.01). The correlation coefficients of the WEMWBS and the SWEMWBS items with the three constructs were consistent with their counterparts at the level of overall WEMWBS and the SWEMWBS scores, and they ranged between low and moderate relationships, as shown in Table 3.

**Table 3:** WEMWBS and SWEMWBS item correlations with other construct-related scales

Items	Needs Satisfaction	Needs Frustration	Depression
MH1^#^	0.437**	− 0.315**	− 0.142**
MH2^#^	0.378**	− 0.310**	− 0.197**
MH3^#^	0.375**	− 0.322**	− 0.331**
MH4	0.217**	− 0.117*	− 0.250**
MH5	0.241**	− 0.176**	− 0.228**
MH6^#^	0.349**	− 0.169**	− 0.231**
MH7^#^	0.280**	− 0.140**	− 0.205**
MH8	0.362**	− 0.339**	− 0.233**
MH9^#^	0.210**	− 0.163**	− 0.214**
MH10	0.440**	− 0.338**	− 0.268**
MH11^#^	0.422**	− 0.221**	− 0.225**
MH12	0.373**	− 0.255**	− 0.291**
MH13	0.352**	− 0.218**	− 0.128**
MH14	0.233**	− 0.210**	− 0.215**
WEMWBS	0.552**	− 0.389**	− 0.373**
SWEMWBS	0.544**	− 0.359**	− 0.337**

*Note*: * *p* < 0.05; ** *p* < 0.01

### Factorial validity of the full-scale and short-scale

Table 4 displays the results of the confirmatory factor analysis performed on the 14-item WEMWBS and the 7-item SWEMWBS. The whole sample was used to conduct CFA for both the full and shortened versions. Model 1 assessed the WEMWBS on its whole scale, with no relationships between measurement errors. Model 1’s findings indicated that the scale did not fit the model well, as indicated by the values of χ^2^ (77) = 462.38, *p* < 0.001, RMSEA = 0.085, TLI = 0.834, CFI = 0.860 and SRMR = 0.057. Some covariances between the error factors reported in the notes of Table 4 were added to Model 2. With χ^2^ (68) = 240.36, *p* < 0.001, RMSEA = 0.061, TLI = 0.916, CFI = 0.937 and SRMR = 0.042, the findings showed good model fit.

**Table 4:** Confirmatory factor analysis of WEMWBS and SWEMWBS

Model	${\varvec{\chi }}^{2}$	*DF*	*p*	CFI	TLI	RMSEA	90% CI for RMSEA	SRMR
***WEMWBS***								
1	462.38	77	0.000	0.860	0.834	0.085	0.078–0.093	0.057
2^*^	240.36	68	0.000	0.937	0.916	0. 061	0.052–0.069	0.042
***SWEMWBS***								
3	69.47	14	0.000	0.874	0.812	0.108	0.084–0.134	0.063
4^**^	15.38	12	0.221	0.992	0.987	0.029	0.000-0.066	0.030

* The adjusted model includes the covariance between the error terms for the items MH1 & MH2, MH3 & MH5, MH6 & MH7, MH10 & MH11, MH8 & MH10, MH5 & MH14, MH3 & MH14, MH5 & MH6, and MH4 & MH12

** Includes the covariance between the error terms for the items MH1 & MH2 and MH6 & MH7

CFA was performed on Model 3 for the shortened version of the 7-item scale without associating the error terms. As the values of χ^2^ (14) = 69.47, *p* < 0.001, RMSEA = 0.108, TLI = 0.812, CFI = 0.874 and SRMR = 0.063 showed, this scale did not meet the cut-off values. Model 4 used the error correlations based on the modification indices to evaluate the SWEMWBS. The values of χ^2^ (12) = 15.38, *p* = 0.221, RMSEA = 0.029, TLI = 0.987, CFI = 0.992 and SRMR = 0.030 showed a good level of model fit. In Model 4, the covariances between the error factors were between MH1 & MH2 and between MH6 & MH6. The results demonstrated that both the WEMWBS and SWEMWBS had a generally satisfactory match for one underlying structure after post hoc adjustment. Also, the SWEMWBS provided a better fit than did the WEMWBS, which was supported by the significant decrease in the chi-square value (∆χ^2^ = 224.98, ∆*df* = 56, *p* < 0.001) and the improvements of other goodness of fit indices RMSEA, TLI, and CFI.

The factor loadings of the items in both WEMWBS and SWEMWBS are presented in Table 5. The factor loadings were greater than 0.40 for the WEMWBS and SWEMWBS items in the three countries and the gender groups. These results also support the construct validity of the full and shortened versions.

**Table 5:** Factor loadings from CFA of WEMWBS and SWEMWBS

Items	WEMWBS					SWEMWBS				
	OM	SA	QA	Male	Female	OM	SA	QA	Male	Female
MH1	0.56	0.51	0.54	0.55	0.61	0.42	0.48	0.43	0.45	0.52
MH2	0.66	0.60	0.64	0.64	0.68	0.60	0.64	0.58	0.61	0.65
MH3	0.64	0.60	0.62	0.57	0.66	0.51	0.61	0.72	0.60	0.60
MH4	0.48	0.50	0.48	0.58	0.43					
MH5	0.60	0.49	0.53	0.59	0.51					
MH6	0.58	0.48	0.54	0.44	0.58	0.60	0.37	0.62	0.45	0.62
MH7	0.57	0.46	0.53	0.46	0.55	0.64	0.40	0.59	0.46	0.63
MH8	0.69	0.61	0.63	0.55	0.72					
MH9	0.58	0.49	0.52	0.63	0.46	0.64	0.46	0.37	0.59	0.43
MH10	0.64	0.49	0.62	0.64	0.53					
MH11	0.60	0.50	0.55	0.54	0.56	0.67	0.56	0.53	0.56	0.64
MH12	0.55	0.51	0.58	0.61	0.48					
MH13	0.54	0.41	0.50	0.50	0.50					
MH14	0.61	0.53	0.52	0.47	0.61					

### Cross-cultural factorial invariance of the full and short scales

For the Arabic versions of the WEMWBS and SWEMWBS, multi-group CFA comparisons were carried out to assess the degree of measurement invariance among the study subgroups. AMOS-22 was used to conduct the series of comparisons, and the outcomes are shown in Table 5.

### Measurement invariance of the WEMWBS

For the configural across-country invariance that was tested in Model A1, the results, χ^2^ (204) = 413.73, *p* < 0.001, RMSEA = 0.039, TLI = 0.900 and CFI = 0.924, indicated acceptable model fit, which demonstrated that the construct can be assumed to be the same across countries. Therefore, the pattern of loadings of items on the latent factor does not differ across cultures. The same results were indicated for configural invariance, as the fit indices were at acceptable levels, χ^2^ (136) = 333.66, *p* < 0.001, RMSEA = 0.046, TLI = 0.908, and CFI = 0.931.

Following testing of the configural invariance, the metric invariance was also tested by constraining the loadings of the items on the constructs to be equivalent in the culture and gender subgroups. For the metric invariance, the factor loadings can be assumed to be the same across countries, and across gender, ∆CFI and ∆RMSEA were found to be acceptable (∆CFI < 0.01 and ∆RMSEA < 0.015).

Additionally, as shown in Table 6 bel, the item intercepts are limited to being equal across the country and gender subgroups to test for scalar invariance. Full scalar invariance across countries and across gender was not caused by the difference between fit indices for scalar invariance, which was slightly outside of acceptable levels for Models A3 and B3 (A3: ∆CFI = − 0.025 and ∆RMSEA = 0.003; B3: ∆CFI = − 0.052 and ∆RMSEA = 0.011). Therefore, partial scalar invariance across cultures and across genders was tested by g the intercepts of items that exhibited a great difference among the subgroups. The intercepts of Items 1, 2, and 12 were found to have the highest change across country groups, and the intercepts of Items 1 and 2 were found to differ across gender subgroups. Therefore, we iteratively relaxed the constraints on intercepts of MH1, MH2, and MH12 to be free across countries and the intercepts of MH1 and MH2 to be free for males and females. For a cross-country subgroup, the improvement in fit indices supports the partial scalar invariance across countries (A3a: ∆CFI = − 0.008 and ∆RMSEA = 0.000). Across gender subgroups, ∆CFI = − 0.012, indicating that partial scalar invariance was not met.

**Table 6:** WEMWBS and SWEMWBS measurement invariance across countries and gender groups

Scales/Level of MI	Model fit indices					Model comparison indices			
	${\chi }^{2}$	*DF*	CFI	TLI	RMSEA	$\varDelta {\chi }^{2}$	*df*	CFI	RMSEA
**WEMWBS**									
**Cross Country**									
A1: Configural	413.73	204	0.924	0.900	0.039	-	-	-	-
A2: Metric	455.28	230	0.919	0.903	0.038	41.55	26	-0.005	-0.001
A3: Scalar	551.86	258	0.894	0.888	0.041	96.58	28	-0.025	0.003
A3a: Partial Scalar (1, 2, 12)	498.66	252	0.911	0.904	0.038	43.38	22	-0.008	0
**Cross Gender**									
B1: Configural	333.66	136	0.931	0.908	0.046	-	-	-	-
B2: Metric	373.05	149	0.922	0.905	0.047	39.39	13	-0.009	0.001
B3: Scalar	534.98	163	0.870	0.855	0.058	161.93	14	-0.052	0.011
B3a: Partial Scalar (1, 2)	442.08	158	0.901	0.886	0.051	69.03	9	-0.021	0.004
**SWEMWBS**									
**Cross Country**									
C1: Configural	30.73	30	0.999	0.998	0.006	-	-	-	-
C2: Metric	54.25	42	0.987	0.981	0.021	23.52	12	-0.012	0.015
C2a: Partial Metric (9)	47.94	40	0.992	0.987	0.017	17.21	10	-0.007	0.011
C3: Scalar	113.94	56	0.940	0.933	0.039	66	16	-0.052	0.022
C3a: Partial Scalar (1, 2)	69.62	50	0.982	0.975	0.024	21.68	10	-0.010	0.007
**Cross Gender**									
D1: Configural	23.36	22	0.999	0.997	0.009	-	-	-	-
D2: Metric	35.88	28	0.992	0.988	0.020	12.52	6	-0.007	0.011
D3: Scalar	86.85	35	0.949	0.939	0.046	50.97	7	-0.043	0.026
D3a: Partial Scalar (1, 2)	57.46	33	0.983	0.970	0.033	21.58	5	-0.009	0.013

### Measurement invariance of the SWEMWBS

The findings of Models C1 and D1 asserted that configural invariance was met for both country and gender subgroups, as indicated by the findings that χ^2^ (204) = 413.73, *p* < 0.001, RMSEA = 0.039, TLI = 0.900 and CFI = 0.924, and χ^2^ (204) = 413.73, *p* < 0.001, RMSEA = 0.039, TLI = 0.900 and CFI = 0.924. Therefore, the pattern of loadings of items on the latent factor does not differ in the three cultures or for males and females.

For the metric invariance, the factor loadings can be assumed to be equal across genders, as the ∆CFI and ∆RMSEA were found to be within the acceptable range (D2: ∆CFI = − 0.007 and ∆RMSEA = 0.011). Moreover, the partial metric invariance was met across country subgroups by relaxing the factor loading of the MH9 to be free across the three countries, which improved the values of the comparison fit indices to fall within the acceptable levels (C2a: ∆CFI = − 0.007 and ∆RMSEA = 0.011).

Additionally, the item intercepts were limited to being equal across the country and gender subgroups to test for scalar invariance. Full scalar invariance across countries and across gender was not met because the difference between fit indices for scalar invariance Models C3 and D3 were out of acceptable levels (C3: ∆FI = − 0.052 and ∆RMSEA = 0.022; D3: ∆CFI = − 0.043 and ∆RMSEA = 0.026). Therefore, partial scalar invariance across cultures and genders was tested by relaxing the intercepts of items that exhibit a great difference among the subgroups. After the intercepts of MH1 and MH2 were relaxed to be free across countries and gender, the results showed an improvement in fit indices that support the partial scalar invariance across the country and gender subgroups (C3a: ∆CFI = − 0.010 and ∆*the* RMSEA = 0.007; D3a: ∆CFI = − 0.009 and ∆RMSEA = 0.013).

## Discussion

The purpose of the current study was to validate the WEMWBS and the SWEMWBS in the context of GCC culture. By analysing the psychometric characteristics of the full and shortened scales using data gathered from Oman, Qatar, and Saudi Arabia, this goal was achieved. We examined the convergent and factorial validity and reliability and investigated the cross-validation of the WEMWBS and the SWEMWBS across genders and the three GCC countries. We chose to introduce covariance between errors in Model 2 for WEMWBS and Model 4 for SWEMWBS because they are related constructs that assess well-being, and they share some common item content. By allowing for covariance between errors, we acknowledge the potential overlap in measurement error that arises from shared question phrasing and thematic content. This approach helps us more accurately capture the unique variance associated with each construct while accounting for common methodological sources of variation. Furthermore, well-being is a multifaceted concept influenced by various interconnected factors, both observable and unobservable. Allowing for covariance between errors recognizes that there may be unmeasured factors or latent variables that affect responses to both WEMWBS and SWEMWBS, such as cultural or contextual influences.

Our findings provide compelling evidence for the convergent validity of the Warwick-Edinburgh Mental Well-being Scale (WEMWBS). We observed a positive relationship between full and short versions of WEMWBS scores and basic needs satisfaction, which is consistent with the scale’s theoretical underpinnings. This alignment with established well-being constructs, as supported by previous work [34] underscores the Arabic version of the WEMWBS’s ability to effectively measure subjective well-being. Furthermore, the negative associations we found between WEMWBS scores and both basic needs frustration and depressive symptoms highlight the scale’s sensitivity to dimensions of mental health that are intimately linked with well-being. This reinforces the idea that the WEMWBS is not only a valuable tool for assessing well-being but also extends its utility to encompass broader dimensions of mental health. The convergence between the WEMWBS and these related constructs underscores its robustness and contributes to the growing body of evidence supporting its validity.

Our findings also revealed that the two Arabic versions yielded results that were as reliable as those from the original and other translated versions of the scale, including the Danish [35], Spanish [31], Italian [36] Norwegian [37], and French [38] versions, according to analyses of internal consistency. The SWEMWBS, in contrast to the WEMWBS’s 14 items, has better factorial validity according to the results of confirmatory factor analysis, which is similar to the results reported in [39]. In addition, there are some similarities between the convergent validity and internal consistency indicators for the WEMWBS and the SWEMWBS scores, supporting those who advocate for the shortened version as an effective instrument for researching the target groups.

Furthermore, in this study, we investigated the measurement invariance of the WEMWBS and the SWEMWBS across three GCC countries. Using the current cross-sectional design, the WEMWBS has satisfactory partial metric invariance across the three countries and partial scalar invariance across genders. This means that the scale measures the same construct reasonably consistently in these countries and can be used to make meaningful comparisons between males and females despite some minor differences in interpretation or response. These findings provide confidence in the scale’s ability to assess mental well-being across different groups and settings in the study. Moreover, the results of measurement invariance of the SWEMWBS have a satisfactory partial scalar invariance across the three countries and two genders. According to the across-country partial metric invariance of the WEMWBS, we can substantiate cross-country comparisons of factor variances and covariances. In addition, by confirming the scalar invariance of SWEMWBS across genders and countries, we can be sure that any statistically significant differences in group means are not caused by variations in scale properties in various groups. This allows us to support multi-group comparisons of factor means (such as t-tests or analysis of variance). In contrast, the WEMWBS can only be used to compare the means of males and females in each country. These findings provided us with sufficient evidence to suggest the utilization of a shortened version of the WEMWBS as an appropriate instrument for evaluating mental well-being across different genders and within three distinct countries.

Lastly, the availability of this validated Arabic version of the Warwick-Edinburgh Mental Well-being Scale (WEMWBS) opens up enormous possibilities for future studies and interventions in the Arab region across males and females. This instrument is essential for assessing and promoting mental well-being by enabling researchers and healthcare practitioners to explore the various factors that influence mental well-being and develop tailored solutions. The accessibility of this resource will help to advance efforts to improve mental well-being in the GCC and other Arab countries.

## Conclusions

Based on the results of our current study, which showed that the Arabic versions of the WEMWBS proved to have appropriate internal consistency, convergence validity, and factorial validity, we conclude that the two instruments are appropriate for use to assess young adults’ mental well-being in Arab countries.

However, there is a need for additional research to determine the reliability of the adaptive measurements regarding the mental well-being of adults in the general population, considering variations in gender, age, socioeconomic status, and prior mental health conditions.

### Limitations

The current study has a few limitations that should be kept in mind when interpreting the findings. An important limitation to highlight is the potential for selection bias, which may arise when the sample does not precisely mirror the broader population in the three countries

Additionally, it should be noted that the sample used in this study was limited to three specific Arab countries and was not representative of all Arab countries. This limitation creates challenges in generalizing the findings to all undergraduate students in Arab countries.

## Data Availability

On reasonable request, the corresponding author will provide access to the data utilized in the current work.

## Abbreviations

WEMWBS: Warwick Edinburgh Mental Well-being Scale
SWEMWBS: Short Warwick Edinburgh Mental Well-being Scale
GCC: The Gulf Cooperation Council
BDI-II: Beck Depression Inventory-II
BPNSFS: Basic Psychological Need Satisfaction-Frustration Scale
